# Comparison of Nutritional Composition and Antioxidant Properties of Pulverized and Unutilized Portions of Waxy Barley

**DOI:** 10.3390/foods12142639

**Published:** 2023-07-08

**Authors:** Tsugumi Furuichi, Daigo Abe, Takuya Uchikawa, Toshihiro Nagasaki, Mina Kanou, Junko Kasuga, Shingo Matsumoto, Yoko Tsurunaga

**Affiliations:** 1 Department of Living Science, Food Science and Nutrition, Tottori College, Tottori 682-8555, Japan; furuichi@cygnus.ac.jp; 2 Western Region Agricultural Research Center, National Agriculture and Food Research Organization, Kagawa 765-8508, Japan; 3 Tottori Institute of Industrial Technology, Tottori 684-0041, Japan; 4 Graduate School of Human and Social Sciences, Shimane University, Shimane 690-8504, Japan; 5 Faculty of Life and Environmental Science, Shimane University, Shimane 690-8504, Japan; 6 Faculty of Human Science, Shimane University, Shimane 690-8504, Japan

**Keywords:** waxy barley, bran, general components, mineral, antioxidant activity, dietary fiber, beta-glucan

## Abstract

To promote the use of waxy barley bran, an underutilized resource, samples of waxy barley were divided into three parts: polished waxy barley powder (PWBP), inner bran layer powder (IBLP), and outer bran layer powder (OBLP). The color and appearance, general properties, minerals, vitamins, β-glucan, antioxidant properties, and aroma of each part were compared. In terms of appearance and color, IBLP and OBLP appeared more yellow than PWBP; general components that were more abundant in IBLP and OBLP compared with PWBP were protein, fat, and ash. IBLP and OBLP had characteristically high values of Mg and Zn, monounsaturated and polyunsaturated fatty acids, vitamin B_1_, total polyphenol content, H-ORAC, and DPPH. In particular, the vitamin B_1_ content of OBLP was approximately 10 times higher than that of PWBP, and Mg and Zn content was more than five times higher than in PWBP. The β-glucan content of IBLP and OBLP was lower than that of PWBP, but relatively high. GC–MS analysis revealed that hexanal was the aroma component common to all three samples, and the peak areas were in the order of PWBP > OBLP > IBLP.

## 1. Introduction

There are many reports on the health benefits of regular barley bran and whole grains. For example, 30 min postprandial serum insulin levels were reduced in rats fed whole grains or bran [1]; many epidemiological studies have reported that the intake of foods containing dietary fiber, especially grains, reduces the risk of chronic diseases [2]. Barley bran has been shown to lower total serum cholesterol in patients with hypercholesterolemia [3]; furthermore, its polyphenolic components can inhibit linoleic acid oxidation and capture superoxide [4]. Based on these findings, waxy barley bran is expected to have similar health benefits, but we have not found any existing reports confirming this. In addition, most of the reports on the functionality and components of barley bran have been based on the studies of the bran itself, and only a few have been on the differences between the inner and outer layers of the bran. For example, McIntosh et al. fed barley bran to rats from different portions of barley grains while examining tumor incidence and found that barley bran containing the aleurone layer was effective at reducing tumor incidence [5,6]. Shimizu et al. fed the inner and outer layers of barley fusuma fractions to rats and evaluated their effects on gastrointestinal tract function and lipid metabolism, and reported that the group that was fed the inner layer showed an increase in fecal weight and a decrease in the pH of fecal content and gastrointestinal transit time [7].

In recent years, the health benefits of the consumption of soluble fiber β-glucan have been recognized worldwide. A daily intake of 5 g of β-glucan significantly reduces serum total cholesterol and LDL cholesterol levels in hypercholesterolemic patients [8] and healthy individuals [9]. β-glucan enhances the efficacy of biological therapy in patients with cancer by promoting the proliferation and activation of peripheral blood monocytes in patients with advanced breast cancer [10]. Furthermore, β-glucan is an active component in lowering postprandial blood glucose levels in patients with type 2 diabetes [11]. Barley is rich in β-glucan [12], and β-glucan content is higher in waxy barley than in normal barley. Due to this high β-glucan content [13], waxy barley cultivars have attracted attention. Among them, the demand for ‘Kirarimochi’, a variety of chewy two-row naked barley, has increased rapidly in recent years owing to its high β-glucan content [14] and chewy texture. In addition, a study on the effects of Kirarimochi consumption on defecation in late-life elderly subjects reported that after 5 months of regular consumption, defecation improved in subjects suffering from constipation [15]. Although barley and waxy barley have been reported to have many functional properties, the pounding yield of barley is approximately 60%, and the remaining 40% of the hull (bran) is disposed after pounding. In Japan, barley is mostly used for the production of beer, shochu, barley tea, etc., and the amount of hulled barley discarded is small. Thus, the hulled portion has not been well-studied. In addition, barley has a unique aroma, and its edible uses, which require pounding, are limited. Thus, research on barley itself has been prioritized over that on the use of the hull. However, it is anticipated that the amount of hulled barley to be discarded will increase as the edible applications of waxy barley expand in the future. Therefore, research on waxy barley hull is highly important for the establishment of a recycling-oriented society. 

Therefore, this study aimed to promote the use of waxy barley bran, which is currently underutilized, and examined the color and appearance, general properties, minerals, vitamins, antioxidant properties, β-glucans, and aroma components of the inner and outer layers of the bran fraction and the pounded grain.

## 2. Materials and Methods

### 2.1. Materials of Barley Variety Kirarimochi

The waxy barley variety Kirarimochi was obtained from a grower in Tottori Prefecture. Barley was milled using a barley milling machine (3RSB-10, HOHDEN Industry Co., Ltd., Kyoto, Japan). The time required for milling 10 kg of raw barley was approximately 4 h. The hulls that emerged during the first 2 h were considered outer bran layer powder (OBLP), and the hulls that emerged during the latter 2 h were considered inner bran layer powder (IBLP). The barley obtained after milling was considered polished waxy barley (PWB). These three components were analyzed. The IBLP and OBLP were used directly as samples as they were in powder form, while the PWB was further ground using an ABSOLUTE MILL (Osaka Chemical Co., Ltd., Osaka, Japan) into polished waxy bran powder (PWBP) and analyzed.

### 2.2. Color Tone

For color tone, L* (lightness degree), a* (red–green degree), and b* (blue–yellow degree) values were measured using a Color Reader (CR-13, Konica Minolta, Inc., Osaka, Japan) on 10 g of the sample spread out in a container, as per the method previously described by Tsurunaga et al. [16]. Three replicates were measured per sample.

### 2.3. Scanning Electron Microscopy (SEM)

SEM microscopy was used to observe the surface structure of the sample powder. The sample was fixed to an SEM specimen stand (Nissin EM Corporation, Type-HM, Tokyo, Japan) with double-sided carbon tape for SEM (Nissin EM Corporation, 8 mm × 20 m, Tokyo, Japan). Gold deposition was carried out, and the specimens were observed via SEM (JSM-IT800SHL, JEOL Ltd., Tokyo, Japan) at an acceleration voltage of 10 kV and a magnification of 600×.

### 2.4. General Components, Vitamin B_1_, and Fatty Acids

Analysis of general components (energy, moisture, protein, fat, carbohydrate, and ash content) was performed by the Shimane Prefectural Environmental Health Corporation, and analysis of vitamin B_1_ and fatty acids was performed by the Japan Food Analysis Center. Methods followed for each component were as follows: protein, Kjeldahl method (protein conversion factor: 6.25); lipid, acid decomposition method; carbohydrate, subtraction method; ash, dry heating method; moisture, normal-pressure heating and drying method. For energy calculation, the following values were used for calculation via the modified Atwater method: protein, 4 kcal/g; fat, 9 kcal/g; and carbohydrates, 4 kcal/g. Vitamin B_1_ was measured using fluorescence detection-high performance liquid chromatography after heat extraction in an acidic aqueous solution. Fatty acids were extracted using a chloroform–methanol mixture, esterified, and measured with hydrogen flame ion detection-gas chromatography.

In addition, the values for general components, vitamin B_1_, and fatty acids for barley-product rice grains (cereals/barley/rice grain barley) and rolled barley (grains/barley/rolled barley/dried) listed in the “Standard Tables of Food Composition in Japan, 2020 edition (8th revision)” [17] are also shown as a comparison target.

### 2.5. Mineral Composition

A powder sample of 0.5 g was mixed with 10 mL of nitrate (Kanto Chemical Co., Inc. Tokyo, Japan), 2 mL of hydrogen peroxide (Kanto Chemical Co., Inc. Tokyo, Japan), and 5 mL of distilled water, then heated at 200 °C for 20 min through the digestion system of ECOPRE-II (ODLAB, Gwangmyeong-si, Korea) six times to obtain the digested solution. Mineral concentrations in the digested solution were analyzed by diluting the digested solution at appropriate rates. Atomic absorption spectrophotometers (Z5010, Hitachi High-Tech Corp., Tokyo, Japan) were used for measurement. The values for mineral content of rice grain barley (cereals/barley/rice grain barley) and rolled barley (cereals/barley/rolled barley/dry), which are barley products listed in the “Standard Tables of Food Composition in Japan, 2020 Edition (8th Revision)” [17], are also shown as a comparison target. 

### 2.6. Vitamin E (Tocopherol)

To 0.5 g of the sample, 2 mL of 10 g/L sodium chloride solution, 0.15 g of pyrogallol, 5 mL of ethanol, and 1 mL of 600 g/L sodium hydroxide solution were added, mixed, and heated in a 70 °C water bath for 30 min. After cooling, 10 mL of 10 g/L sodium chloride solution and 7 mL of hexane: 2-propanol: ethyl acetate (9:1.5:1 *v*/*v*/*v*) mixture were added, and the mixture was extracted by shaking for 5 min. After centrifugation at 1500 rpm for 5 min (20 °C) (Model 6200, KUBOTA Corporation Co., Ltd., Tokyo, Japan) and separation of the organic solvent layer, 7 mL of hexane: 2-propanol: ethyl acetate mixture was added. The same procedure was repeated twice, and all organic solvent layers were combined and removed under reduced pressure using a rotary evaporator (RE300, Yamato Scientific Co., Ltd. Tokyo, Japan). A hexane solution (5 mL) of 0.1 g/L ethoxyquin was added to the sample for analysis. The samples were analyzed using an HPLC system (LC-20AD, Shimazu Co., Ltd., Kyoto, Japan) by comparison with standard compounds using normal-phase column (YMC-Pack SIL, SIL-06, S-5, 4.6 × 250 mm, Sigma-Aldrich, St. Louis, MO, USA). The analysis conditions for fluorescence detection RF-10AXL (Shimazu Co., Ltd.) were as follows: excitation wavelength, 298 nm; measurement wavelength, 325 nm; column oven temperature, 40 °C; flow rate, 1.5 mL/min; mobile phases, hexane: 2-propanol: acetic acid (1000:6:5 *v*/*v*/*v*) containing 5 µg/mL butylated hydroxytoluene injection volume, 20 µL.

### 2.7. Determination of Total Polyphenol Content (TPC) and Antioxidant Activity

Sample solutions were prepared by mixing 0.5 g of each sample powder with 10 mL of 60% ethanol and extracting at 40 °C, 150 rpm for 2 h in a stirring water bath (BW201, Daiwa Kagaku K.K., Tokyo, Japan) [16,18]. This extract solution was also used to measure antioxidant properties. Extraction was performed twice per treatment. TPC was determined using the Folin–Ciocalteu method as previously described by Tsurunaga et al. [16]. TPC was expressed as mg equivalent/100 g, using CTN as a standard (mg CTN eq/100 g). Six replicates of each measurement were performed. Two methods were used for antioxidant activity. DPPH radical scavenging activity was measured according to the method described by Tsurunaga et al. [19] using stable DPPH radicals and expressed as Trolox equivalents (µmol TE/g). Each measurement was performed in six replicates. H-ORAC was measured according to the method described by Watanabe et al. [20]. H-ORAC values were expressed as Trolox equivalents (µmol TE/g). Each measurement was performed in three replicates.

### 2.8. Beta-Glucan

Quantitative determination of β-glucan was performed via the McCleary method (enzymatic method) using a β-glucan assay kit (K-BGLU, Megazyme, Bray, Ireland). The analytical procedure was partially modified from the method described in the kit, as previously described by Yoo et al. [21]. A total of 0.1 g of each sample was mixed with 1 mL of ethanol (50%, *v*/*v*) and then with 9 mL of sodium phosphate buffer (20 mM, pH 6.5). The resulting mixture was then incubated in a boiling water bath for 2 min before being removed, stirred with a vortex mixer, and heated for another 1 min. After incubation, the sample was cooled to 50 °C and 0.2 mL of lichenase solution (10 U) was added and incubated (50 °C, 60 min, stirred every 15 min). Following this, 30 mL of distilled water was added to this mixture and the resulting mixture was stirred and centrifuged (5800, KUBOTA Corporation Co., Ltd., Tokyo, Japan) at 3000 rpm for 10 min. After centrifugation, 0.2 mL of each supernatant was transferred to three 5 mL tubes. After incubation of the three tubes at 50 °C for 18 min, glucose oxidase/peroxidase reagent (3.3 mL) was added and incubated at 50 °C for 24 min. After incubation for 30 min at room temperature, 300 µL from each tube was dispensed into a 96-well plate, and the absorbance was measured at 510 nm using a Microplate Reader (EPOCH-S, BioTek Instruments Inc., Winooski, Vermont, USA). Extraction was performed twice per treatment. Each measurement was performed in duplicate.

### 2.9. GC–MS Analysis

Flavor components were measured using the method previously described by Tsurunaga et al. [16], which is a modification of Farneti’s method [22].

### 2.10. Statistical Processing

Statistical processing was performed using SPSS (Ver.28, IBM Inc, Chicago, IL, USA), and after one-way ANOVA, Tukey’s HSD method was used to test for significance at the 5% level.

## 3. Results and Discussion

### 3.1. Appearance, Color Tone, and SEM

**Figure 1:**
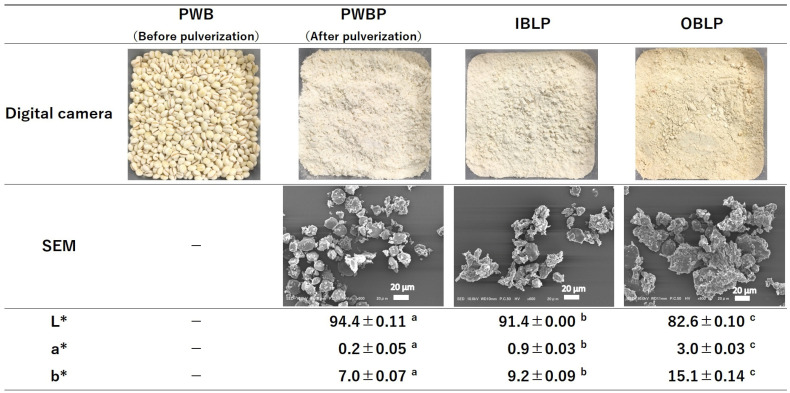
Digital camera images, SEM images, and color differences in ‘Kirarimochi’. PWB: polished waxy barley, PWBP: polished waxy barley powder, IBLP: inner bran layer powder, OBLP; outer bran layer powder. The values are presented as means and standard errors (*n* = 3). The different letters (a–c) indicate statistical differences (*p* < 0.05).

Digital camera images, SEM images, and color results are shown in Figure 1; PWB was slightly brown, while PWBP was bright white; IBLP and OBLP were slightly more yellowish than PWBP powder, and OBLP was cream-colored (Figure 1). SEM images showed that PWBP had many rounded starch grains; IBLP and OBLP also had starch grains, but there were also many irregularly shaped and sized (15–60 µm) objects. The irregularly shaped and sized objects were considered to be bran parts (specifically, the pericarp and seed coat), and more than half the content of both IBLP and OBLP was presumed to be bran. The order of L* values, indicating brightness, of the different components was as follows: PWBP > IBLP > OBLP, with significant differences (*p* < 0.05). In contrast, the order of a* (red–green) and b* (yellow–blue) values were as follows: OBLP > IBLP > PWBP, with significant differences (*p* < 0.05). These results were consistent with the macroscopic (digital camera image) results. There was concern that if IBLP and OBLP were to be used in food products in the future, it would affect appearance, which is considered important in terms of quality. However, this experiment showed that both IBLP and OBLP were light in color, suggesting that they have little effect on the foods to which they are added and can be used in a variety of foods. 

### 3.2. General Composition

Table 1 shows the general composition of waxy barley. Comparisons were also made with the barley products (rice grain barley and rolled barley) listed in the Standard Tables of Food Composition in Japan, 2020 edition (8th revision) [17]. Rice grain barley is barley that has been processed to look and feel like rice and is eaten as barley rice; rolled barley is barley that has been steamed and crushed with a pressure roller to make it easier to cook with rice. Comparing the PWBP of waxy barley and barley products, energy (PWBP, 367 kcal/100 g; rice grain barley, 333 kcal/100 g; rolled barley, 329 kcal/100 g) and carbohydrate (PWBP, 82.8 g/100 g; rice grain barley, 76.2 g/100 g; rolled barley, 78.3 g/100 g) were higher in PWBP. However, protein (PWBP, 6.5 g/100 g; rice grain barley, 7.0 g/100 g; rolled barley, 6.7 g/100 g) and fat (PWBP, 1.1 g/100 g; rice grain barley, 2.1 g/100 g; rolled barley, 1.5 g/100 g) tended to be slightly higher in barley products. Next, comparing the PWBP and bran portion of waxy barley, the energy content was in the following order: OBLP (402 kcal/100 g) > IBLP (370 kcal/100 g) > PWBP (367 kcal/100 g). Similar trends were observed for protein (OBLP, 14.9 g/100 g; IBLP, 11.4 g/100 g; PWBP, 6.5 g/100 g), fat (OBLP, 9.5 g/100 g; IBLP, 4.5 g/100 g; PWBP, 1.1 g/100 g), and ash (OBLP, 4.1 g/100 g; IBLP, 2.6 g/100 g; PWBP, 0.8 g/100 g). On the other hand, carbohydrate content showed the opposite trend (PWBP, 82.8 g/100 g; IBLP, 71.0 g/100 g; OBLP, 64.1 g/100 g). It is generally known that the bran portion of cereals is high in protein, fat, and mineral components [23,24]. Specifically, the bran portion of wheat is reported to contain 4.3 g/100 g of fat, 15.6 g/100 g of protein, and 0.47 g/100 g of ash, compared with the endosperm portion, which is reported to contain 1.0 g/100 g of fat, 10.3 g/100 g of protein, and 5.8 g/100 g of ash, approximately 1.3–12 times higher. The bran portion of oats contains 7.0 g/100 g of fat, 17.3 g/100 g of protein, and 2.89 g/100 g of ash, while whole oats have 6.3 g/100 g of fat, 13.2 g/100 g of protein, and 1.75 g/100 g of ash, approximately 1–1.6 times higher. It has been reported that for barley, the removal of the outer layer decreases protein, lipid, and ash content and increases starch and β-glucan content [25], and it was confirmed that waxy barley follows the same trend as these cereals.

### 3.3. Minerals

The Na, K, Ca, Mg, Fe, Zn, Cu, and Mn content is shown in Table 2. First, comparing the PWBP of waxy barley and barley products, the K content was higher in barley products (rice grain barley, 170 mg/100 g; rolled barley, 210 mg/100 g) than in waxy barley PWBP (63 mg/100 g). Mg content was slightly higher in rolled barley (40 mg/100 g) than in waxy barley PWBP (39 mg/100 g). Other minerals were higher in waxy barley, especially trace minerals, which were much higher in PWBP (Fe, 4.2 mg/100 g; Zn, 9.3 mg/100 g; Cu, 7.84 mg/100 g; Mn, 10.65 mg/100 g) than in barley products. Next, comparing PWBP and bran portions, the Cu content was higher in PWBP (7.84 mg/100 g) than in bran (IBLP, 4.37 mg/100 g; OBLP, 7.09 mg/100 g) (no significant difference, *p* > 0.05), but other mineral content tended to be higher in bran than in PWBP. For minerals other than Ca and Cu, the order was significantly higher for OBLP > IBLP > PWBP (*p* < 0.05). In particular, Mg (PWBP, 39 mg/100 g; OBLP, 354 mg/100 g) and Zn (PWBP, 9.3 mg/100 g; OBLP, 50.3 mg/100 g) were approximately five times higher in OBLP than in PWBP. The barley products contained higher amounts of K, but other minerals were more abundant in waxy barley, particularly in the bran portion, confirming its usefulness as a source of minerals. According to the Food Composition Table of the US Department of Agriculture, Agricultural Research Service [26] (data not displayed), the Na content of wheat meal and bran is 2 mg/100 g for both meal and bran, but K (150 mg/100 g for meal and 1180 mg/100 g for bran), Ca (15 mg/100 g for meal and 73 mg/100 g for bran), Mg (22 mg/100 g for meal and 611 mg/100 g for bran), Fe (1.2 mg/100 g for meal and 611 mg/100 g for bran), Zn (0.7 mg/100 g for meal and 7.3 mg/100 g for bran), Cu (0.21 mg/100 g for meal and 1.00 mg/100 g for bran), Mn (0.82 mg/100 g for meal and 11.50 mg/100 g for bran), which range from 4.7 to 27.8 times higher in the bran portion than in the refined flour. Comparisons of the mineral content of whole grain and bran of oats have shown that while the Na and Cu content is higher in the whole grain than in the bran, other mineral content was 1 to 1.9 times higher in the bran. Compared with these cereals, waxy barley is particularly rich in trace minerals, and the bran portion has higher mineral content than the endosperm portion, which is consistent with the ash results mentioned in Section 3.2. K intake, which promotes urinary excretion of Na, is important for Japanese people because their average Na intake is higher than that of other countries. Fe intake is slightly less than the target amount (men: 3000 mg/day or more; women: 2600 mg/day or more) as per the Dietary Reference Intakes for Japanese (2020 version) [27]. In addition, Fe is an important constituent of hemoglobin and various enzymes, and its deficiency leads to anemia and a decrease in motor function, cognitive efficacy, etc. Furthermore, loss of Fe due to menstrual blood and increased demand during pregnancy and lactation have a significant impact on the required dietary Fe intake. According to the 2019 National Health and Nutrition Survey [28], the Fe intake of Japanese people (aged 20 years or older) is 8.3 ± 3.3 mg/day (men) and 7.5 ± 3.2 mg/day (women). According to the Dietary Reference Intakes for Japanese (2020 version) [27], the recommended amounts are 7.5 mg/day (men) and 10.5 mg/day (for menstruating women), and inadequate Fe intake is observed especially among women. Since the bran portion of waxy barley contains minerals necessary for Japanese people, its utilization instead of disposal will improve national mineral intake status. 

### 3.4. Fatty Acid Content

The fatty acid content of waxy barley is shown in Table 3. Myristic acid, pentadecanoic acid, palmitoleic acid, arachidic acid, eicosenoic acid, behenic acid, docosenoic acid, and tetracosenoic acid were not detected in PWBP. The fatty acid content of PWBP and barley products (rice grain barley and rolled barley) was compared and found to be similar. Rice grain barley fatty acid content was 1.3–2.5 times higher in most fatty acids than PWBP, but PWBP had a higher stearic acid content (0.03 g/100 g) than rice grain barley (0.025 g/100 g). The total fatty acid content was as follows: OBLP (8.18 g/100 g), IBLP (3.98 g/100 g), and PWBP (1.15 g/100 g), in an order consistent with that of the fat content of these components. Linoleic acid (4.4 g/100 g), α-linolenic acid (0.42 g/100 g), and eicosenoic acid (0.09 g/100 g) content was 2–3 times higher than in IBLP. Linoleic acid (n-6, 18:2) and α-linolenic acid (n-3, 18:3), both fatty acids that are abundant in the bran of waxy barley, are essential fatty acids, which cannot be synthesized in the body and whose deficiency results in dermatitis. Intake of n-6 and n-3 fatty acids has been reported to contribute to the improvement of dyslipidemia and diabetes. For dyslipidemia, replacing saturated fatty acids with n-6 fatty acids has been reported to lower total cholesterol and LDL cholesterol [29]. Intake of n-3 fatty acids has also been reported to reduce total mortality, myocardial infarction mortality, and sudden death in patients with coronary heart disease to cardiovascular disease [30]. For diabetes, increased intake of α-linolenic acid [31] and linoleic acid [32] have been reported to reduce the risk of developing diabetes. As per the 2019 National Health and Nutrition Survey in Japan [28], the intake of n-6 fatty acids for Japanese people (aged 20 years and older) was 11.61 ± 5.75 g/day (men) and 9.84 ± 5.14 g/day (women). For n-3 fatty acids, the daily intake was 2.68 ± 1.84 g/day (men) and 2.27 ± 1.63 g/day (women). According to the Dietary Reference Intakes for Japanese (2020 version) [27], the recommended intake of n-6 fatty acids is at least 10 g/day (men) and 8 g/day (women), and that of n-3 fatty acids is at least 2.0 g/day (men) and 1.6 g/day (women), which is more or less in compliance. In any case, waxy barley bran is expected to be used as a source of n-6 and n-3 fatty acids to reduce the risk of dyslipidemia and diabetes, as mentioned above.

### 3.5. β-Glucan

**Figure 2:**
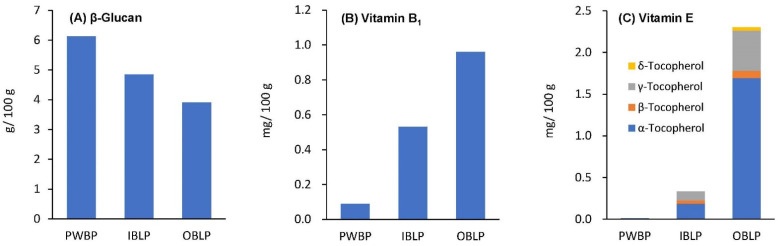
Differences in β-glucan (**A**), vitamin B_1_ (**B**), and vitamin E (**C**) content in ‘Kirarimochi’ at different degrees of milling. Bars in β-glucan (**A**) and vitamin E (**C**) represent the mean value (*n* = 2). Analysis of vitamin B_1_ (**B**) was performed by Japan Food Research Laboratories.

The β-glucan content (shown in Figure 2) tended to be highest in PWBP (6.13 ± 0.034 g/100 g), followed by IBLP (4.85 ± 0.062 g/100 g) and OBLP (3.91 ± 0.017 g/100 g). It was confirmed that β-glucan is abundant in the cell wall of the endosperm of barley [33] but is also relatively abundant in the bran portion of the barley. β-glucan is a type of soluble dietary fiber that can be isolated from algae, fungi, and mushrooms as well as cereals. Depending on the source, its properties, such as glycosidic linkages, degree of branching, molecular weight, and solubility, vary, but β-glucan from cereals is mainly a mixture of β (1→3) and β (1→4) glucans [34]. Cereal-derived β-glucans have been reported to have health benefits, such as lowering postprandial blood glucose levels after oral glucose loading in patients with type 2 diabetes [11], reducing serum total and LDL cholesterol [8,9], and reducing the risk of coronary heart disease [35]. Similar effects can be expected from β-glucan derived from waxy barley. In this study, the β-glucan content of waxy barley bran was higher than that of oats (1.79–3.33 g/100 g) [36] and rye (1.9–2.5 g/100 g) observed in previous studies [37], suggesting that waxy barley is a rich source of β-glucan. 

### 3.6. Vitamin B_1_

Vitamin B_1_ content in waxy barley (shown in Figure 2) was as follows: OBLP, 0.96 mg/100 g; IBLP, 0.53 mg/100 g; PWBP, 0.09 mg/100 g. Vitamin B_1_ is found in the bran portion of cereals [38], and the same holds true for waxy barley. The intake of vitamin B_1_ by Japanese people (aged 20 years or older) as per the 2019 National Health and Nutrition Survey in Japan [28] was 1.03 ± 0.50 mg/day (men) and 0.88 ± 0.42 mg/day (women), which exceeded the recommended amount (men: 0.5 mg/day, women: >0.5 mg/day) detailed in the Dietary Reference Intakes for Japanese People (2020 edition) [27]. Vitamin B_1_ is a water-soluble vitamin and is involved in the metabolism of glucose and branched amino acids as a coenzyme-type ThDP. Vitamin B_1_ deficiency causes neuroinflammation and brain tissue damage. Vitamin B_1_ deficiency includes beriberi and Wernicke–Korsakoff syndrome. Vitamin B_1_ deficiency is not uncommon in cases of severe malnutrition, but is rare in healthy people in developed countries. However, Ozawa et al. [39] suggest that vitamin B_1_ deficiency should be considered in the differential diagnosis of normal-living patients with congestive heart failure and that the dietary education of the population should be improved to ensure adequate vitamin B_1_ intake.

### 3.7. Vitamin E

Vitamin E (tocopherols) in waxy Barley is shown in Figure 2. Tocopherol exists in the form of four homologues (α, β, γ, and δ) in the chemical structure. Total vitamin E content was highest in OBLP (2.30 mg/100 g), followed by IBLP (0.33 mg/100 g), followed by PWBP (0.01 mg/100 g). β, γ, and δ-tocopherol isomers were not detected in PWBP, and δ was not detected in IBLP. α, γ, β, and δ were detected in OBLP in that order. It has been reported that about 80% of tocopherols are contained in the germ fraction [40]. The present experiment is also in agreement with this report, as tocopherols were abundant in IBLP and OBLP, which contain the germ fraction. Barley tocopherols tend to have a higher content of α-tocopherol [41], and the variety, Kirarimochi, used in this experiment had a similar trend.

### 3.8. TPC, H-ORAC, and DPPH Value

**Figure 3:**
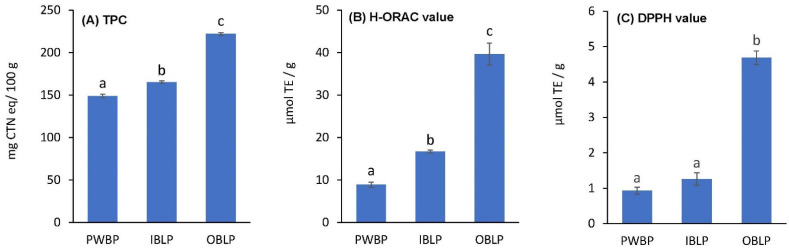
Antioxidant activity of ‘Kirarimochi’ at different degrees of milling. Antioxidant activity was evaluated as the TPC (**A**), H-ORAC (**B**), and DPPH (**C**) radical scavenging activity. PWBP: polished waxy barley powder, IBLP: inside bran layer powder, OBLP: outside bran layer powder, TPC: total polyphenol content, CTN eq: catechin equivalent, TE: Trolox equivalent. Bars represent the mean ± standard error (TPC and DPPH were *n* = 12, H-ORAC was *n* = 6). The different letters (a–c) indicate statistical differences (*p* < 0.05).

TPC, H-ORAC, and DPPH values are shown in Figure 3. TPC was significantly higher (*p* < 0.05) in OBLP (221.9 ± 1.6 mg CTN eq/100 g), followed by IBLP (165.2 ± 1.4 mg CTN eq/100 g) and PWBP (148.8 ± 2.0 mg CTN eq/100 g) (*p* < 0.05). It has been reported that the TPC value of barley increases from the center to the outer layers [42], and similar results were observed in waxy barley. The H-ORAC values were significantly higher for OBLP (39.6 ± 2.6 µmol TE/g), followed by IBLP (16.7 ± 0.4 µmol TE/g) and PWBP (8.9 ± 0.6 µmol TE/g) (*p* < 0.05). Furthermore, DPPH values were highest in OBLP (4.7 ± 0.2 µmol TE/g), followed by IBLP (1.3 ± 0.2 µmol TE/g) and PWBP (0.9 ± 0.1 µmol TE/g), but there was no significant difference between PWBP and IBLP; only OBLP showed significantly higher H-ORAC values. The DPPH value of barley is also reported to be higher in the outer layer and lower in the inner layer [43], and the same was confirmed for waxy barley. The H-ORAC and DPPH values showed a similar trend to that of TPC, confirming the trend of higher antioxidant properties from the components of the inner layers to the outer layers, i.e., in terms of TPC, H-ORAC, and DPPH values, PWBP < IPLB < OBLP. Regarding the antioxidant properties, OBLP showed two to four times higher H-ORAC values and 3.6 times higher DPPH values than IBLP and PWBP, indicating that OBLP has greater antioxidant properties than IBLP and PWBP. Barley bran contains polyphenols such as flavanols (catechins, procyanidins, prodelphinidins), flavonols (quercetin), and hydroxy silicic acids (ferulic acid, caffeic acid, coumaric acid), which have been reported as the strongest contributors to antioxidant capacity [42,44]. In particular, flavanols have been studied in human dietary interventions using flavanol-containing foods to dilate peripheral blood vessels [45] for the improvement of insulin resistance and glucose tolerance [46], in addition to their beneficial effects on coronary circulation by acutely increasing coronary flow velocity reserve [47], and other health benefits have been reported. The high antioxidant capacity of IBLP and OBLP in waxy barley may be due to the involvement of these components. However, the polyphenol content of the samples was not determined in this experiment and is a subject for future work. In this experiment, among the many methods for measuring antioxidant properties, only H-ORAC and DPPH methods were used. In the DPPH method, after the radical scavenging reaction, the quinone structure is changed to the oxidized form. The quinone structure is reduced in the presence of a polar solvent like ethanol, which regenerates the catechol structure; it has been reported that it reacts with DPPH radicals again [48]. Therefore, it is necessary for future studies to conduct analysis using the 2,2’-azinobis (3-ethylbenzothiazoline-6-sulfonic acid (ABTS)) method, which is less susceptible to the effect of catechol structure regeneration.

### 3.9. GC–MS

**Figure 4:**
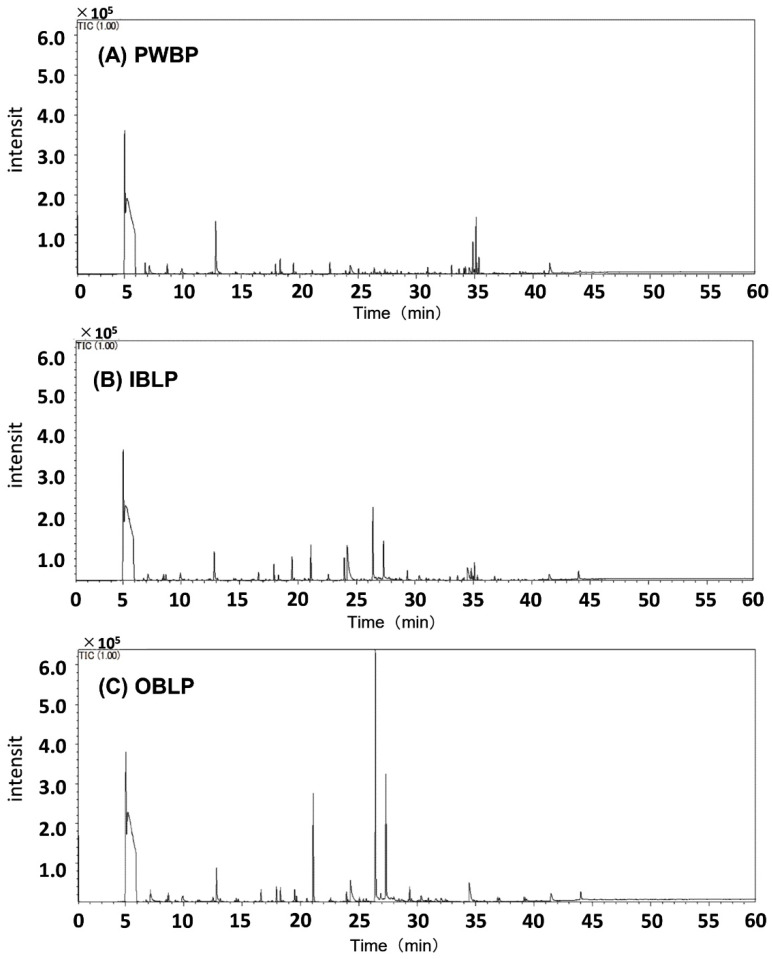
Gas chromatography–mass spectrometry chromatograms of PWBP (**A**), IBLP (**B**), and OBLP (**C**). PWBP: polished waxy barley powder, IBLP: inner bran layer powder, OBLP: outer bran layer powder.

**Figure 5:**
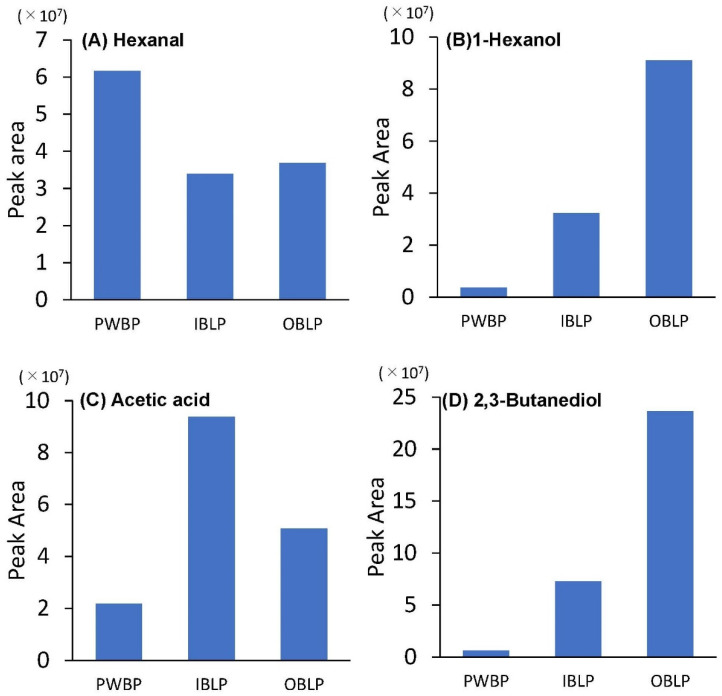
Peak areas of aroma compounds in ‘Kirarimochi’ at different degrees of milling (*n* = 1). The aroma components are (**A**) Hexanal, (**B**) 1-Hexanol, (**C**) Acetic acid, and (**D**) 2,3-Butanediol. PWBP: polished waxy barley powder, IBLP: inner bran layer powder, OBLP: outer bran layer powder.

The chromatograms of the samples are shown in Figure 4. First, the common peak at around 13 min was presumed to be hexanal. Hexanal is the component that gives soybeans their foul odor [49,50] and has also been reported as a major aroma component of rice [51], barley, and wheat [52]. The peaks characteristic of IBLP and OBLP were estimated to be 1-hexanol at 21 min, acetic acid at 24 min, and 2,3-butanediol at 26 min. 1-hexanol is considered a green note compound and has been reported as an aroma component in Chinese tea [53] and barley [52]. Acetic acid is a type of carboxylic acid with a strong pungent odor found in vinegar and has been reported as an aroma component of cooked barley [54], while 2,3-butanediol is a divalent alcohol, is a volatile component of cocoa beans [55] and black Thai rice [56], and imparts a sweet aroma. Hexanal was estimated to be a common aroma component of waxy barley. It was thought that lipoxygenase and hydroperoxide isomerase contained in barley oxidize unsaturated fatty acids to produce volatile components [52]. The amount of unsaturated fatty acids was higher in IBLP and OBLP (Table 3), but the peak area of hexanal in GC–MS tended to be higher in PWBP (Figure 5). In soybean milk, a positive correlation between odorant content and the content of bean lipids, linoleic acid, and lipoxygenase has been reported [50]. The addition of antioxidants such as phenolic compounds to processed oats has been found to inhibit hexanal formation [57]. The peak area of hexanal was higher in PWBP than in OBLP or IBLP in waxy barley because of the higher polyphenol content and antioxidant capacity in the bran portion, which inhibited enzymatic oxidation of polyunsaturated fatty acids. In OBLP and IBLP, the peak areas of alcohols and acids such as 1-hexanol, acetic acid, and 2,3-butanediol tended to be higher, indicating a difference in composition between PWBP and aroma components (Figure 5). Aroma is an important factor involved in the taste of food, but the bran portion of barley and cereals is said to be a limiting factor in consumption because it imparts a unique flavor [58]. The results of this experiment showed that the peak area of hexanal tended to be lower in OBLP and IBLP than in PWBP, indicating that waxy barley bran could be made acceptable to consumers through masking and other measures during cooking and processing. However, in this study, the values were calculated using only peak areas without standard reagents, the number of measurement repetitions was only one, and the fragrance components were only estimated. We believe that these issues require further study.

## 4. Conclusions

In this study, waxy barley was divided into three parts (PWBP, IBLP, and OBLP) to determine the differences between them. The color tone of IBLP and OBLP was yellowish compared with that of PWBP, but their light color suggested that the effect of processing waxy barley on the visual appearance of food was small. It was also found that the most abundant nutrients in the bran portion (IBLP and OBLP) were energy, protein, fat, ash, vitamin B_1_, and vitamin E. The β-glucan content of IBLP and OBLP was lower than that of PWBP, but still relatively high. The polyphenol content, H-ORAC, and DPPH values were all highest in OBLP, confirming that the polyphenol content was proportional to the antioxidant capacity. Hexanal was estimated as a common aroma component in waxy barley, but its abundance in the bran portion tended to be low. Appearance and aroma are important factors in determining the quality of food, and this study showed that waxy barley bran has the potential to be accepted by consumers. It is also rich in minerals, β-glucan (soluble dietary fiber), and antioxidants, all of which Japanese people tend to lack, making it a material that can contribute to improving the health of Japanese people. This study had some limitations. Only the total polyphenol content of each barley fraction was measured and the detailed types of polyphenols were not estimated. Since several polyphenols have been identified in barley bran, a detailed analysis of the different polyphenols in waxy barley should also be identified and their effects on antioxidant capacity analyzed. The aroma component was also measured only once, using a simple analytical method. We believe that quantitative measurement and identification of aroma components are important for processing and promoting the consumption of waxy barley bran.

## Figures and Tables

**Table 1:** General composition of ‘Kirarimochi’ at different degrees of milling and processed barley products.

	Waxy Barley *^1^			Rice Grain Barley *^2^	Rolled Barley *^2^
	PWBP	IBLP	OBLP		
Energy (kcal/100 g)	367	370	402	333	329
Moisture (g/100 g)	8.8	10.5	7.4	14.0	12.7
Protein (g/100 g)	6.5	11.4	14.9	7.0	6.7
Fat (g/100 g)	1.1	4.5	9.5	2.1	1.5
Carbohydrate (g/100 g)	82.8	71.0	64.1	76.2	78.3
Ash (g/100 g)	0.8	2.6	4.1	0.7	0.7

PWBP: polished waxy barley powder, IBLP: inner bran layer powder, OBLP: outer bran layer powder. *^1^: Analysis of general components was performed by the Shimane Prefectural Environmental Health Corporation. *^2^: Values for rice grain barley and rolled barley were taken from Standard Tables of Food Composition in JAPAN–2020–(Eighth Revised Edition).

**Table 2:** Mineral content of ‘Kirarimochi’ at different degrees of milling and processed barley products.

	Waxy Barley			Rice Grain Barley *^1^	Rolled Barley *^1^
	PWBP	IBLP	OBLP		
Na (mg/100 g)	6.29 ± 0.09 ^a^	12.22 ± 0.23 ^b^	12.04 ± 0.34 ^b^	2	2
K (mg/100 g)	62.93 ± 0.86 ^a^	122.25 ± 2.28 ^b^	120.43 ± 3.39 ^b^	170	210
Ca (mg/100 g)	30.09 ± 6.21	30.31 ± 0.23	59.67 ± 14.30	17	21
Mg (mg/100 g)	39.13 ± 0.78 ^a^	181.17 ± 0.35 ^b^	353.75 ± 6.15 ^c^	25	40
Fe (mg/100 g)	4.18 ± 0.04 ^a^	7.46 ± 0.08 ^b^	10.52 ± 0.12 ^c^	1.2	1.1
Zn (mg/100 g)	9.26 ± 0.36 ^a^	26.76 ± 0.06 ^b^	50.27 ± 1.06 ^c^	1.2	1.1
Cu (mg/100 g)	7.84 ± 3.57	4.37 ± 0.13	7.09 ± 0.12	0.37	0.22
Mn (mg/100 g)	10.65 ± 0.21 ^a^	14.51 ± 0.09 ^b^	30.16 ± 0.96 ^c^	―	0.86

*^1^: A Standard Tables of Food Composition in JAPAN–2020–(Eighth Revised Edition). PWBP: polished waxy barley powder, IBLP: inner bran layer powder, OBLP: outer bran layer powder. The values for waxy barley fractions represent means and standard errors (*n* = 3). The different letters (a–c) indicate statistical differences (*p* < 0.05).

**Table 3:** Fatty acid content of ‘Kirarimochi’ at different degrees of milling and processed barley products.

	Waxy Barley *^1^			Rice Grain Barley *^2^	Rolled Barley *^2^
	PWBP	IBLP	OBLP		
Total fatty acids (g/100 g)	1.15	3.89	8.18	1.69	1.18
Saturated fatty acids (g/100 g)	0.40	0.94	1.83	0.58	0.43
Monounsaturated fatty acids (g/100 g)	0.08	0.69	1.53	0.20	0.13
Polyunsaturated fatty acids (g/100 g)	0.67	2.26	4.82	0.91	0.62
Myristic acid (g/100 g)	―	―	0.02	0.008	0.006
Pentadecanoic acid (g/100 g)	―	―	0.01	0.001	0.001
Palmitic acid (g/100 g)	0.37	0.90	1.70	0.53	0.39
Palmitoleic acid (g/100 g)	―	―	0.10	0.001	0.001
Stearic acid (g/100 g)	0.03	0.04	0.06	0.025	0.022
Oleic acid (g/100 g)	0.08	0.63	1.37	―	0.099
Linoleic acid (g/100 g)	0.64	2.12	4.40	0.86	0.59
Alpha-linolenic acid (g/100 g)	0.03	0.14	0.42	0.054	0.033
Arachidic acid (g/100 g)	―	―	0.02	0.002	0.002
Eicosenoic acid (g/100 g)	―	0.03	0.09	0.010	0.006
Behenic acid (g/100 g)	―	―	0.02	0.007	0.002
Docosenoic acid (g/100 g)	―	0.03	0.05	0.017	0.011
Tetracosenoic acid (g/100 g)	―	―	0.01	0.000	0.002

PWBP: polished waxy barley powder, IBLP: inner bran layer powder, OBLP: outer bran layer powder. *^1^: Analysis of fatty acids was performed by Japan Food Research Laboratories. *^2^: Values for rice grain barley and rolled barley were taken from Standard Tables of Food Composition in JAPAN–2020–(Eighth Revised Edition).

## Data Availability

Data are contained within the article.

## References

[1] Harding S.V., Storsley J., Thandapilly S.J., Ames N.P. (2013). Lower 30 Minute Serum Insulin in Healthy Sprague-Dawley Rats Consuming Chips from Specific Barley Flour Blends. Cereal Chem..

[2] Ye E.Q., Chacko S.A., Chou E.L., Kugizaki M., Liu S. (2012). Greater whole-grain intake is associated with lower risk of type 2 diabetes, cardiovascular disease, and weight gain. J. Nutr..

[3] Lupton J.R., Robinson M.C., Morin J.L. (1994). Cholesterol-lowering effect of barley bran flour and oil. J. Am. Diet. Assoc..

[4] Tamagawa K., Iizuka S., Fukushima S., Endo Y., Komiyama Y. (1997). Antioxidative activity of polyphenol extracts from barley bran. J. Jpn. Soc. Soc. Food Sci. Technol..

[5] McIntosh G., Jorgensen L., Royle P. (1993). The potential of an insoluble dietary fiber-rich source from barley to protect from DMH-induced intestinal tumors in rats. Nutr. Canc..

[6] McIntosh G.H., Le Leu R.K., Royle P.J., Young G.P. (1996). A comparative study of the influence of differing barley brans on DMH-induced intestinal tumours in male Sprague-Dawley rats. J. Gastroenterol. Hepatol..

[7] Shimizu J., Tamagawa K., Ikeda A., Naganuma K., Wada M., Takita T., Innami S. (2001). Effects of Different Fractions of Barley Bran on Gastrointestinal Function and Lipid Metabolism in Rats. J. Jpn. Assoc. Diet. Fiber Res..

[8] Theuwissen E., Mensink R.P. (2007). Simultaneous intake of β-glucan and plant stanol esters affects lipid metabolism in slightly hypercholesterolemic subjects. J. Nutr..

[9] Naumann E., Van Rees A.B., Önning G., Öste R., Wydra M., Mensink R.P. (2006). β-Glucan incorporated into a fruit drink effectively lowers serum LDL-cholesterol concentrations. Am. J. Clin. Nutr..

[10] Demir G., Klein H., Mandel-Molinas N., Tuzuner N. (2007). Beta glucan induces proliferation and activation of monocytes in peripheral blood of patients with advanced breast cancer. Int. Immunopharmacol..

[11] Tapola N., Karvonen H., Niskanen L., Mikola M., Sarkkinen E. (2005). Glycemic responses of oat bran products in type 2 diabetic patients. Nutr. Metab. Cardiovasc. Dis..

[12] Zhang G., Junmei W., Jinxin C. (2002). Analysis of β-glucan content in barley cultivars from different locations of China. Food. Chem..

[13] Ullrich S., Clancy J., Eslick R., Lance R. (1986). β-Glucan content and viscosity of extracts from waxy barley. J. Cereal Sci..

[14] Yanagisawa T., Nagamine T., Takahashi A., Takayama T., Doi Y., Matsunaka H., Fujita M. (2011). Breeding of Kirari-mochi: A new two-rowed waxy hull-less barley cultivar with superior quality characteristics. Breed. Sci..

[15] Taniguchi K., Komae K., Takahashi A., Yoshioka T., Sone Y. (2017). Effect of waxy barley, Kirarimochi, consumption on bowel movements of late-stage elderly residents at Roken nursing home. J. Physiol. Anthropol..

[16] Tsurunaga Y., Kanou M., Ikeura H., Makino M., Oowatari Y., Tsuchiya I. (2022). Effect of different tea manufacturing methods on the antioxidant activity, functional components, and aroma compounds of *Ocimum gratissimum*. LWT.

[17] The Subdivision on Resources, The Council for Science and Technology Ministry of Education, Culture, Sports, Science and Technology, Japan (2020). Standard Tables of Food Composition in Japan.

[18] Katsube T., Tabata H., Ohta Y., Yamasaki Y., Anuurad E., Shiwaku K., Yamane Y. (2004). Screening for antioxidant activity in edible plant products: Comparison of low-density lipoprotein oxidation assay, DPPH radical scavenging assay, and Folin−Ciocalteu assay. J. Agr. Food Chem..

[19] Tsurunaga Y., Takahashi T., Katsube T., Kudo A., Kuramitsu O., Ishiwata M., Matsumoto S. (2013). Effects of UV-B irradiation on the levels of anthocyanin, rutin and radical scavenging activity of buckwheat sprouts. Food Chem..

[20] Watanabe J., Oki T., Takebayashi J., Yamasaki K., Takano-Ishikawa Y., Hino A., Yasui A. (2012). Method validation by interlaboratory studies of improved hydrophilic oxygen radical absorbance capacity methods for the determination of antioxidant capacities of antioxidant solutions and food extracts. Anal. Sci..

[21] Yoo H.U., Ko M.J., Chung M.S. (2020). Hydrolysis of beta-glucan in oat flour during subcritical-water extraction. Food Chem..

[22] Farneti B., Cristescu S.M., Costa G., Harren F.J., Woltering E.J. (2012). Rapid tomato volatile profiling by using proton-transfer reaction mass spectrometry (PTR-MS). J. Food Sci..

[23] Seal C.J., Courtin C.M., Venema K., de Vries J. (2021). Health benefits of whole grain: Effects on dietary carbohydrate quality, the gut microbiome, and consequences of processing. Compr. Rev. Food Sci. Food Saf..

[24] Carcea M. (2021). Value of Wholegrain Rice in a Healthy Human Nutrition. Agriculture.

[25] Baik B.K., Ullrich S.E. (2008). Barley for food: Characteristics, improvement, and renewed interest. J. Cereal Sci..

[26] FoodData Central. https://fdc.nal.usda.gov./.

[27] Ministry of Health, Labour and Welfare (2020). The Dietary Reference Intakes for Japanese.

[28] Ministry of Health, Labour and Welfare (2019). The National Health and Nutrition Survey in Japan.

[29] Mensink R.P., Zock P.L., Kester A.D.M., Katan M.B. (2003). Effects of dietary fatty acids and carbohydrates on the ratio of serum total to HDL cholesterol and on serum lipids and apolipoproteins: A meta-analysis of 60 controlled trials. Am. J. Clin. Nutr..

[30] Bucher H.C., Hengstler P., Schindler C., Meier G. (2002). N-3 polyunsaturated fatty acids in coronary heart disease: A meta-analysis of randomized controlled trials. Am. J. Med..

[31] Brostow D.P., Odegaard A.O., Koh W.P., Duval S., Gross M.D., Yuan J.M., Pereira M.A. (2011). Omega-3 fatty acids and incident type 2 diabetes: The Singapore Chinese Health Study. Am. J. Clin. Nutr..

[32] Wang L., Folsom A.R., Zheng Z.J., Pankow J.S., Eckfeldt J.H., Investigators A.S. (2003). Plasma fatty acid composition and incidence of diabetes in middle-aged adults: The Atherosclerosis Risk in Communities (ARIC) Study. Am. J. Clin. Nutr..

[33] Langenaeken N.A., Ieven P., Hedlund E.G., Kyomugasho C., Van De Walle D., Dewettinck K., Van Loey A.M., Roeffaers M.B.J., Courtin C.M. (2020). Arabinoxylan, β-glucan and pectin in barley and malt endosperm cell walls: A microstructure study using CLSM and cryo-SEM. Plant J..

[34] Kaur R., Sharma M., Ji D.W., Xu M., Agyei D. (2020). Structural Features, Modification, and Functionalities of Beta-Glucan. Fibers.

[35] Bernstein A.M., Titgemeier B., Kirkpatrick K., Golubic M., Roizen M.F. (2013). Major Cereal Grain Fibers and Psyllium in Relation to Cardiovascular Health. Nutrients.

[36] Gujral H.S., Sharma P., Rachna S. (2011). Effect of sand roasting on beta glucan extractability, physicochemical and antioxidant properties of oats. LWT.

[37] Ragaee S.M., Campbell G.L., Scoles G.J., McLeod J.G., Tyler R.T. (2001). Studies on rye (*Secale cereale* L.) lines exhibiting a range of extract viscosities. 1. Composition, molecular weight distribution of water extracts, and biochemical characteristics of purified water-extractable arabinoxylan. J. Agric. Food Chem..

[38] Lebiedzinska A., Szefer P. (2006). Vitamins B in grain and cereal-grain food, soy-products and seeds. Food Chem..

[39] Ozawa H., Homma Y., Arisawa H., Fukuuchi F., Handa S. (2001). Severe metabolic acidosis and heart failure due to thiamine deficiency. Nutrition.

[40] Falk J., Krahnstover A., van der Kooij T.A.W., Schlensog M., Krupinska K. (2004). Tocopherol and tocotrienol accumulation during development of caryopses from barley (*Hordeum vulgare* L.). Phytochemistry.

[41] Panfili G., Fratianni A., Di Criscio T., Marconi E. (2008). Tocol and beta-glucan levels in barley varieties and in pearling by-products. Food. Chem.

[42] Gangopadhyay N., Harrison S.M., Brunton N.P., Hidalgo-Ruiz J.L., Gallagher E., Rai D.K. (2018). Brans of the roller-milled barley fractions rich in polyphenols and health-promoting lipophilic molecules. J. Cereal Sci..

[43] Madhujith T., Izydorczyk M., Shahidi F. (2006). Antioxidant Properties of Pearled Barley Fractions. J. Agric. Food. Chem..

[44] Gangopadhyay N., Rai D.K., Brunton N.P., Gallagher E., Hossain M.B. (2016). Antioxidant-guided isolation and mass spectrometric identification of the major polyphenols in barley (*Hordeum vulgare*) grain. Food Chem..

[45] Fisher N.D.L., Hughes M., Gerhard-Herman M., Hollenberg N.K. (2003). Flavanol-rich cocoa induces nitric-oxide-dependent vasodilation in healthy humans. Am. J. Hypertens..

[46] Grassi D., Lippi C., Necozione S., Desideri G., Ferri C. (2005). Short-term administration of dark chocolate is followed by a significant increase in insulin sensitivity and a decrease in blood pressure in healthy persons. Am. J. Clin. Nutr..

[47] Schroeter H., Heiss C., Balzer J., Kleinbongard P., Keen C.L., Hollenberg N.K., Sies H., Kwik-Uribe C., Schmitz H.H., Kelm M. (2006). (-)-Epicatechin mediates beneficial effects of flavanol-rich cocoa on vascular function in humans. Proc. Natl. Acad. Sci. USA.

[48] Sendra J.M., Sentandreu E., Navarro J.L. (2007). Kinetic model for the antiradical activity of the isolated p-catechol group in flavanone type structures using the free stable radical 2, 2-diphenyl-1-picrylhydrazyl as the antiradical probe. J. Agric. Food Chem..

[49] Kobayashi A., Wang D., Yamazaki M., Tatsumi N., Kubota K. (2000). Aroma constituents of tofu (soy bean curd) contributing to its flavor character. J. Jpn. Soc. Food Sci. Technol..

[50] Yuan S.H., Chang S.K.C. (2007). Selected odor compounds in soymilk as affected by chemical composition and lipoxygenases in five soybean materials. J. Agric. Food Chem..

[51] Yang D.S., Shewfelt R.L., Lee K.S., Kays S.J. (2008). Comparison of odor-active compounds from six distinctly different rice flavor types. J. Agric. Food Chem..

[52] Cramer A.C.J., Mattinson D.S., Fellman J.K., Baik B.K. (2005). Analysis of volatile compounds from various types of barley cultivars. J. Agric. Food. Chem..

[53] Qin Z.H., Pang X.L., Chen D., Cheng H., Hu X.S., Wu J.H. (2013). Evaluation of Chinese tea by the electronic nose and gas chromatography-mass spectrometry: Correlation with sensory properties and classification according to grade level. Food Res. Int..

[54] Takemitsu H., Amako M., Sako Y., Kita K., Ozeki T., Inui H., Kitamura S. (2019). Reducing the undesirable odor of barley by cooking with superheated steam. J. Food Sci. Technol. Mysore.

[55] Rodriguez-Campos J., Escalona-Buendia H.B., Orozco-Avila I., Lugo-Cervantes E., Jaramillo-Flores M.E. (2011). Dynamics of volatile and non-volatile compounds in cocoa (*Theobroma cacao* L.) during fermentation and drying processes using principal components analysis. Food. Res. Int..

[56] Ajarayasiri J., Chaiseri S. (2008). Comparative study on aroma-active compounds in Thai, black and white glutinous rice varieties. Agric. Nat. Resourc..

[57] Viscidi K.A., Dougherty M.P., Briggs J., Camire M.E. (2004). Complex phenolic compounds reduce lipid oxidation in extruded oat cereals. LWT.

[58] Heiniö R.L., Noort M.W.J., Katina K., Alam S.A., Sozer N., De Kock H.L., Hersleth M., Poutanen K. (2016). Sensory characteristics of wholegrain and bran-rich cereal foods–A review. Trends Food Sci. Technol..

